# Effects of omecamtiv mecarbil and mavacamten in isolated human atrium

**DOI:** 10.1007/s00210-022-02333-0

**Published:** 2022-11-18

**Authors:** Lina Maria Rayo Abella, Christian Höhm, Britt Hofmann, Ulrich Gergs, Joachim Neumann

**Affiliations:** 1 Institute for Pharmacology and Toxicology, Medical Faculty, Martin Luther University Halle-Wittenberg, Magdeburger Str. 4, D-06097 Halle, Germany; 2 Cardiac Surgery, Medical Faculty, Martin Luther University Halle-Wittenberg, D-06097 Halle, Germany

**Keywords:** Omecamtiv, Mavacamten, Mouse atrium, Human atrium, Force of contraction

## Abstract

Heart failure is a syndrome that can result from impaired heart muscle contractions like in dilative cardiomyopathy but also from hypertrophic obstructive cardiomyopathy (HOCOM). A pharmacological therapy might lie in Ca^2+^-sensitizing or Ca^2+^-desensitizing drugs, respectively. Such drugs are thought to be omecamtiv mecarbil (OME) and mavacamten (MYK-461), respectively. Their function in contracting human muscle is not fully understood and was the focus of the present study. OME from 1 nM to 10 µM cumulatively applied failed to raise force of contraction in human right atrial preparations strips (HAP) or mouse left atrial preparations (LA). However, OME prolonged time to peak tension and time of relaxation in HAP and LA but did not alter the beating rate in right atrial preparations from mice (RA). In contrast, MYK-461 (10 nM to 10 µM) reduced concentration- and time-dependently force of contraction in HAP and LA. MYK-461 (10 µM) did not affect the beating rate in RA. In summary, the present data failed to detect an increase in force of contraction for OME, in human and mouse atrium. In contrast, a Ca^2+^ desensitizer studied for comparison was able to reduce force of contraction in HAP and LA. We conclude that putative beneficial effects of OME in dilated cardiomyopathy cannot be explained by positive inotropic effects in the HAP, whereas beneficial functional effects of MYK-461 in HOCOM can be explained by negative inotropic effects in HAP.

**Supplementary Information:**

The online version contains supplementary material available at 10.1007/s00210-022-02333-0.

## Introduction

Systolic heart failure is a potentially deadly disease syndrome. The current drug treatment of this disease syndrome remains unsatisfactory (review: Ahmad et al. 2019). Hence, new drugs with novel mechanisms of action are continuously being studied. Few new inotropes show positive results in clinical trials (review: Ahmad et al. 2019). One such novel compound, studied in patients with systolic heart failure, is omecamtiv mecarbil (OME) (Teerlink et al. 2016, 2020, Fig. 1). However, the mechanism(s) of action of OME remain(s) the subject of ongoing debate and experiments. OME was found in screening for myosin activators in vitro (Malik et al. 2011). OME turned out to increase the sensitivity of mouse skinned cardiac fibers (myocytes, where the sarcolemma has been removed) to Ca^2+^ (Malik et al. 2011). This concept of Ca^2+^ sensitization itself is not novel but has been studied for four decades (Solaro and Rüegg 1982). One early Ca^2+^ sensitizer was CGP 48,506. CGP 48,506 increased force of contraction in guinea pig cardiac preparations and isolated ventricular muscle strips from the human heart (Neumann et al. 1996, Zimmermann et al. 1996). Interestingly, the positive inotropic effect of CGP 48,506 was accompanied by a prolonged duration of force generation in single muscle contractions (Neumann et al. 1996, Zimmermann et al. 1996). It was already feared at that time that the prolongation of force generation might make the patients (those with high pulse rates) susceptible to contractures (increases in diastolic muscle tension) by impairing relaxation (Neumann et al. 1996, Zimmermann et al. 1996). A next such Ca^2+^ sensitizer then came out with levosimendan: but the consensus seems to shift to the view that levosimendan might act mainly as a phosphodiesterase inhibitor (Maack et al. 2019, Orstavik et al. 2014, Endoh 2015). OME has been reported to raise contractility under auxotonic conditions (beating without pre-load or after-load in isolated electrically driven rat ventricular cardiomyocytes: Malik et al. 2011, Nagy et al. 2015) and isolated electrically driven canine ventricular cardiomyocytes (Shen et al. 2010). In sharp contrast, others found omecamtiv to reduce force of contraction in isolated rat papillary muscle under isometric conditions (Lookin et al. 2022) or to leave force of contraction unaltered in isolated muscle preparations from human atrium or failing (heart transplant recipients) human ventricle (Dashwood et al. 2021). As far as we know, OME has not yet been studied in isolated mouse electrically driven left atrial preparations or spontaneously beating mouse right atrial preparations, which was investigated in the present study. The present study in mice atria might be of potential mechanistically relevance, as many transgenic and knockout mice are now available and could be used as tools to understand the mechanism(s) of action of OME better. Moreover, in human atrial preparations, one could hypothesize that in the presence of OME, the response of force of contraction to extracellular Ca^2+^ in intact muscle strips might be potentiated. Such an approach has been used by others to study whether or not levosimendan acted as a Ca^2+^ sensitizer in isolated rat papillary muscles (Orstavik et al. 2014). However, such data are currently not available for OME but will be presented here for mice and human atrium.

**Fig. 1:**
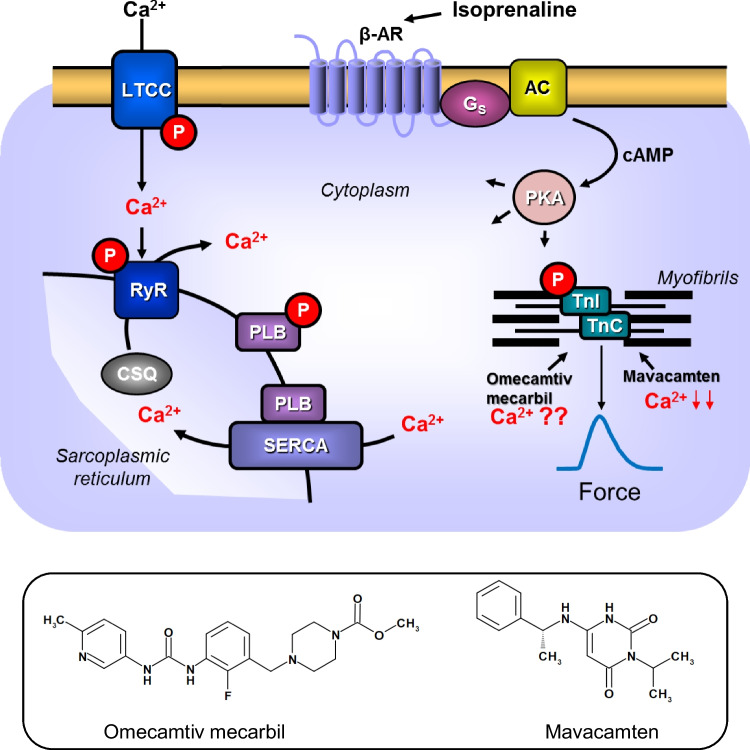
Scheme: Potential mechanism(s) of action of omecamtiv mecarbil and mavacamten-461 in the human cardiomyocytes. Stimulation of the activity of β-adrenoceptors (β-AR) by isoprenaline leads via stimulatory GTP-binding proteins (G_s_) to an increase of adenylyl cyclase (AC) activity. Adenylyl cyclase increases the formation of 3′,5′-cyclic adenosine monophosphate (cAMP) that stimulates cAMP-protein kinase (PKA). PKA phosphorylates and thus activates inter alia phospholamban (PLB) at the amino acid serine 16, the inhibitory subunit of troponin (TnI), the ryanodine receptor (RYR), and the L-type calcium channel (LTCC). Calcium cations (Ca^2+^) are stored on calsequestrin (CSQ) in the sarcoplasmic reticulum and are released via RYR from the sarcoplasmic reticulum (SR). These released Ca^2+^ bind to troponin C (TnC) on thin myofilaments, and as a result, systolic force is augmented. In cardiac diastole, Ca^2+^ concentrations fall because Ca^2+^ is pumped into the SR via the SR-calcium ATPase (SERCA). The activity of SERCA is increased when phospholamban is phosphorylated on amino acid serine 16. Negative inotropic effects can be used to treat HOCOM (hypertrophic obstructive cardiomyopathy) like propranolol which blocks β-adrenoceptors or verapamil which inhibits the LTCC. Alternatively, force can be reduced by compounds that reduce the Ca^2+^ affinity of myofilaments like mavacamten (structural formula, bottom). Systolic heart failure due to, e.g., idiopathic cardiomyopathy, can be treated by compounds that increase cytosolic Ca^2+^ levels like cAMP-increasing agents (phosphodiesterase inhibitor, β-adrenoceptor agonists). At constant Ca^2+^ levels force of contraction can be increased by Ca.^2+^ sensitizers, like omecamtiv mecarbil (structural formula, bottom) or CGS 48506

Other causes of heart failure are genetically caused hypertrophies of the ventricular muscle. The mutations often occur in myosin and are often first manifested in the interventricular septum. This cardiac hypertrophy of the septum can lead to signs and symptoms reminiscent of aortic valve stenosis like angina pectoris. These septal hypertrophies can be treated, in principle, by removing surgically or chemically (septal injection of pure ethanol) parts of the inimical muscle mass in the ventricle. A less invasive treatment option is the use of negative inotropic drugs. The assumption being that negative inotropic drugs like propranolol or verapamil thereby reduce muscular mechanical obstruction in the outflow tract of the left ventricle and thus facilitate left ventricular ejection of blood. Here, the opposite approach to OME has entered the clinic: a compound named mavacamten (MYK-461) (Fig. 1) was developed as a Ca^2+^ desensitizer (Green et al. 2016). In other words, MYK-461 desensitizes the myofilaments to Ca^2+^ and thereby reduces force of contraction and therefore has been studied to treat hypertrophic obstructive cardiomyopathy (HOCOM). Like OME, also MYK-461 was also found by using an in vitro screen of myosin function, by the same company (Green et al. 2016). MYK-461 reduced the contractility of isolated electrically driven rat ventricular cardiomyocytes and led to a half maximal reduction in cell wall motion at a concentration of 0.18 µM MYK-461 (Green et al. 2016). MYK-461 was early on studied in living mice with a genetic point mutation in myosin that led to a phenotype reminiscent of hypertrophy in human HOCOM (Green et al. 2016). In living wild-type mice, the authors described a reduction of ejection fraction (using echocardiography) by MYK-461 (Green et al. 2016). Based on these data in mouse and subsequent data in larger animals (cats: Stern et al. 2016), first clinical trials were performed in HOCOM which were not unsuccessful because the symptoms of patients like angina pectoris were decreased (Olivotto et al. 2020). Though symptomatic relief occurred, no decrease of mortality has yet been reported (Olivotto et al. 2020). To the best of our knowledge, MYK-461 has never been studied in mouse atrial preparations or in isolated human atrial muscle strips.

Hence, we wanted to compare two drugs acting oppositely on cardiac myosin function/Ca^2+^ sensitivity of myofilaments and used already in study patients with heart failure. One was presumably a negative inotropic drug (MYK-461) and the other a putative positive inotropic drug (OME). We wanted to know how they affect beating rate in mouse atrium and force of contraction in mouse atrium and human atrium in the organ bath. These studies were hoped to lead us to better clinical use of such drugs acting on Ca^2+^ sensitivity in syndromes of human heart failure.

## Materials and methods

### Contractile studies in mice

In brief, wild-type mice were sacrificed, the thorax was opened, and the heart was mobilized and cut from the ascending aorta to make sure the right atrium was not damaged. Then the whole heart was transferred to a dissection chamber filled with gassed Tyrode’s solution at room temperature. Right or left atrial preparations were isolated and mounted in organ baths as described by Gergs et al. (2013, 2017, 2019a) and Neumann et al. (2003). Force was detected under isometric conditions, amplified and transmitted to a digitizer, and quantified by commercial software (LabChart 8, ADInstruments, Spechbach, Germany).

### Contraction studies in human atrium

These experiments were performed as reported repeatedly (e.g., Gergs et al. 2009, 2019b; Neumann et al. 2021b). In brief, during cardiac surgery, at the site where the cannula for extracorporeal circulation entered the heart, small muscle strips were obtained from the right atrium. Patients were aged between 56 and 78 years. Medication included acetylsalicylic acid, nitrates, diuretics, β-adrenoceptor blockers, and anticoagulants. Atrial trabeculae were dissected and mounted in organ bath and electrically stimulated (1 Hz) and processed like mouse preparations (see above).

### Data analysis

Data were treated as in most our previous studies (e.g., Gergs et al. 2019a; Neumann et al. 2021a, b). Shown are means ± standard error of the mean. Statistical significance was estimated by Student’s t-test. A *P*-value of less than 0.05 was considered significant. Experimental data for agonist-induced positive inotropic and chronotropic effects were analyzed by fitting sigmoidal curves to the experimental data with GraphPad Prism 5.0 (GraphPad Software, San Diego, CA, USA). All other statistical analyses were performed as indicated in the figures and tables.

### Drugs and materials

Stock solutions were freshly prepared daily. Mavacamten (6-[[(1S)-1-phenylethyl]amino]-3-propan-2-yl-1H-pyrimidine-2,4-dione or MYK-461) were purchased from Cayman Chemical (Ann Arbor, Michigan 48,108, USA). Omecamtiv mecarbil (methyl 4-[(2-fluoro-3-{[N-(6-methylpyridin-3-yl)-carbamoyl]-amino}-phenyl)methyl]piperazine-1-carboxylate or CK-1827452)) was purchased from Selleck Chemicals, Berlin, Germany. (-)-Isoprenaline ( +)-bitartrate was purchased from Sigma-Aldrich (Taufkirchen, Germany). All other chemicals were of the highest purity grade commercially available. Deionized water was used throughout the experiments.

## Results

### Effect of omecamtiv mecarbil and mavacamten-461 on beating rate and force of contraction in mouse right atrial preparations

OME effects on force of contraction of spontaneously beating mouse right atrial preparations (RA) were qualitatively similar as in left atrial preparations (LA, see below). Cumulatively applied OME (10 nM–10 µM) did not increase force of contraction in isolated spontaneously beating RA (data not shown). Moreover, under conditions where OME (10 nM–10 µM) prolonged time parameters and reduced the rate of tension development in LA, OME failed to alter the spontaneous beating rate in RA (Table 1). Similar to OME, MYK-461 failed to alter beating rate in RA (Table 1). As a positive control, isoprenaline (1 µM) in the same samples increased the beating rate (Table 1).

**Table 1:** Effects of omecamtiv mecarbil and mavacamten-461 on left and right mouse atrial preparations

Parameters	Maximal effect (10 µM) in % of Ctr (*n* = 6)	Isoprenaline effect (1 µM) in % of Ctr (*n* = 3–4)
Omecamtiv mecarbil		
Force of contraction (LA)	85.8 ± 8.9	248.1 ± 24.5*
Beating rate (RA)	95.1 ± 3.0	170.5 ± 10.6*
Time to peak tension (LA)	113.2 ± 1.9*	88.1 ± 0.5*
Time of relaxation (LA)	572.4 ± 85.1*	62.6 ± 3.8*
dF/dt min (LA)	54.1 ± 5.9*	477.3 ± 69.4*
dF/dt max (LA)	76.6 ± 7.8*	276.9 ± 28.1*
Mavacamten-461		
Force of contraction (LA)	41.0 ± 5.1*	499.0 ± 85.6
Beating rate (RA)	117.8 ± 5.5	171.2 ± 8.5*
Time to peak tension (LA)	97.9 ± 0.6*	105.0 ± 15.1
Time of relaxation (LA)	93.2 ± 1.5*	83.9 ± 3.6
dF/dt min (LA)	41.9 ± 5.7*	348.4 ± 126.3
dF/dt max (LA)	40.5 ± 5.4*	333.8 ± 121.1

*p < 0.05 versus pre-drug value (Ctr) set as 100%. *LA*, left atrium; *RA*, right atrium

### Effect of omecamtiv mecarbil and mavacamten-461 on force of contraction in electrically driven mouse left atrial preparations and electrically driven human right atrial preparations

**Fig. 2:**
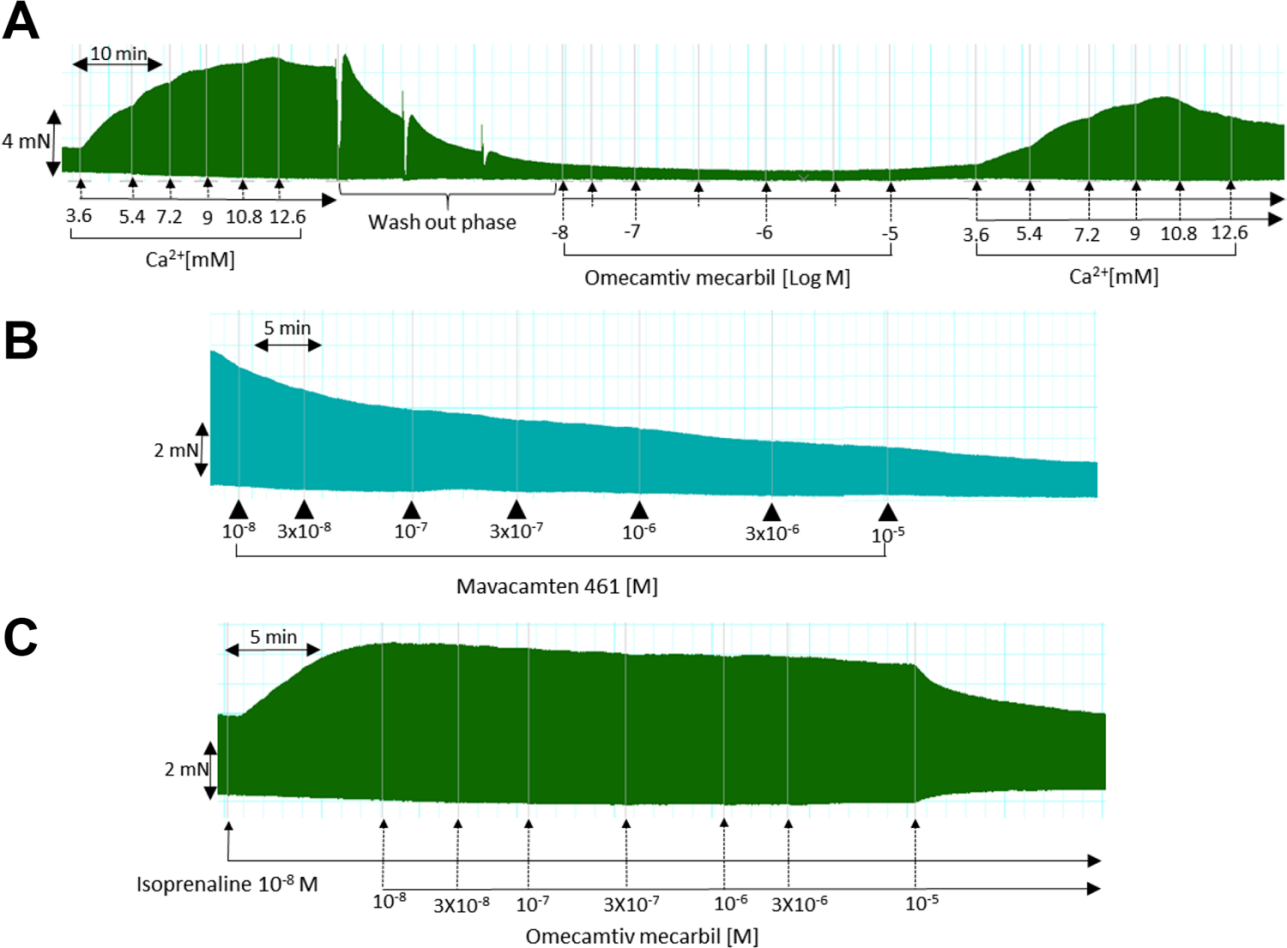
Original recordings of force of contraction of paced human right atrial preparations. **A** No positive inotropic effect of omecamtiv mecarbil was noted in human atrium. First, calcium ions (Ca^2+^) were cumulatively applied. Then washout was preformed thrice. Thereafter, omecamtiv mecarbil (10 nM to 10 µM) was cumulatively applied. Additionally, without washout, Ca^2+^ was applied again. **B** A negative inotropic effect of mavacamten-461 was noted in human atrium. Mavacamten-461 (10 nM to 10 µM) was cumulatively applied. **C** Antiadrenergic effects of omecamtiv mecarbil in human atrium. First, 10 nM isoprenaline was applied. When a plateau was reached, OME (10 nM to 10 µM) was cumulatively applied. Note that under these conditions, OME clearly exhibits an antiadrenergic negative inotropic effect. Vertical bars indicate force in millinewton (mN). Horizontal bars indicate incubation times in minutes (min)

It is known that Ca^2+^ sensitizers like EMD 57,033 increase the potency of Ca^2+^ in the organ bath to increase force of contraction (e.g., Orstavik et al. 2014). Therefore, we likewise constructed concentration–response curves for Ca^2+^ in mouse left atrial preparations and human right atrial preparations (original recording: Fig. 2A) in the absence and thereafter in the presence of cumulative applied OME (10 nM to 10 µM). It turned out that this curve was not shifted by OME to the left as expected for a Ca^2+^ sensitizer (original recording: Fig. 2A). Moreover, OME under these conditions reduced and thus did not increase force of contraction, as might be expected from the literature (Fig. 2A).

MYK-461 concentration- and time-dependently reduced force of contraction in isolated electrically driven human right atrial preparations (original recording: Fig. 2B). As a positive control, subsequently applied 1 µM isoprenaline increased force of contraction (Table 1).

Moreover, we wanted to know whether in the presence of isoprenaline (when cytosolic Ca^2+^ is elevated as schematically shown in Fig. 1), OME might increase force of contraction what would be expected if OME really is a Ca^2+^-sensitizing agent, as reported in the literature. This was not the case. After stimulation of force of contraction in human right atrial preparations, isoprenaline (10 nM) increased force, but additionally and subsequently applied OME did not increase force of contraction, but clearly at 10 µM, OME reduced force of contraction (Fig. 2C). The same results were seen in muscle strips from two other patients. Of note here is that we used 10 nM isoprenaline that is about at the EC_50_ value (half maximally effective concentration) for isoprenaline in human atrium. This was done to ensure that a positive inotropic effect of OME would still be measurable as isoprenaline at 10 nM did not stimulate force maximally. This finding may have a clinical bearing. It might indicate that at the beginning of heart failure when noradrenaline levels in patient plasma are high and β-adrenergic stimulation is presumably elevated, OME might be especially detrimental for cardiac force generation.

### Effect of omecamtiv mecarbil on time parameters of single contractions in electrically driven mouse left atrial preparations and electrically driven human right atrial preparations

**Fig. 3:**
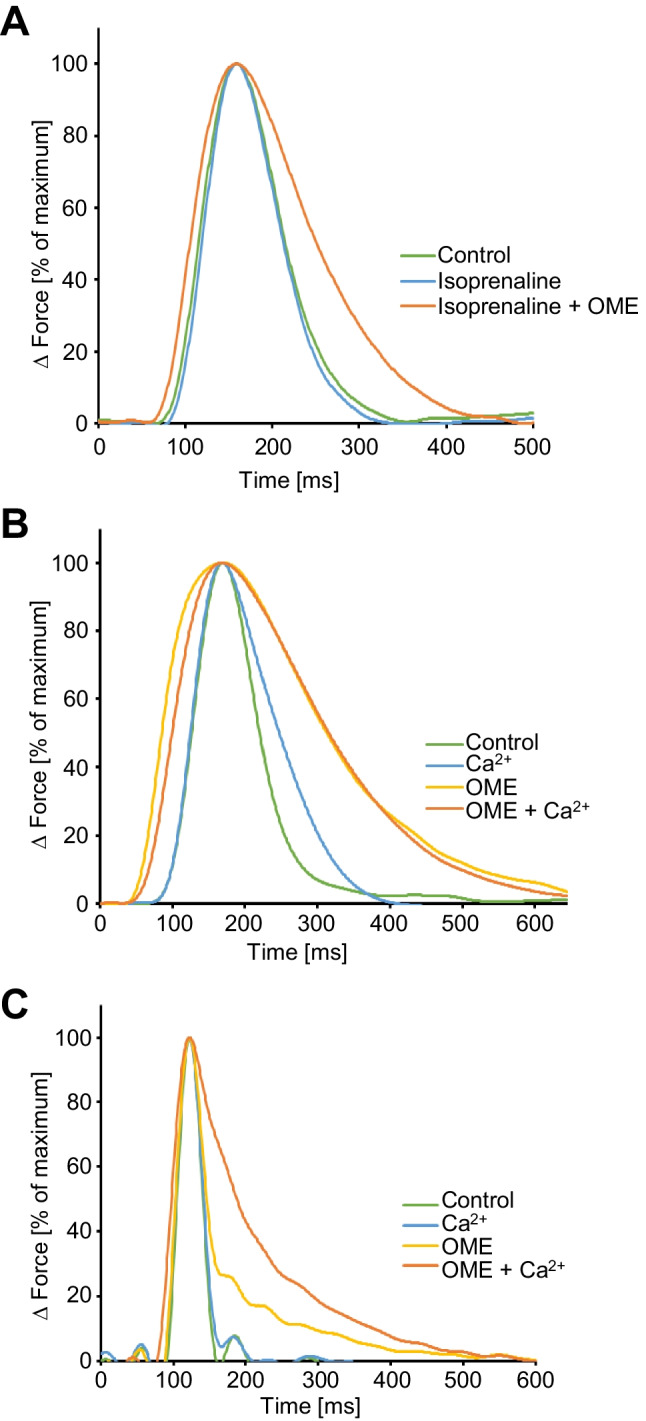
Effects of omecamtiv mecarbil (OME) on time parameters of contraction in human and mouse atrial preparations. OME prolongs the duration of contraction. Ordinates: normalized force of contraction (% of maximum effect). Abscissae: time of contraction in milliseconds (ms). **A** After control contractions in human atrial preparations (Ctr), isoprenaline (10 nM) shortened the duration of contraction, and additionally applied OME prolonged duration of contraction. **B** In contrast, in human atrial preparations, calcium ions (Ca^2+^) prolonged duration of contraction that was much more prolonged by additionally applied OME. **C** Similarly, in mouse left atrial preparations, OME prolonged duration of contraction alone and even more in the additional presence of Ca^2+^

As seen in Fig. 2A, inotropy in the human atrial muscle was not increased by OME: however, we noted that OME concentration- and time-dependently prolonged time to peak tension and time of relaxation in mouse left atrial preparations (Fig. 3C) but more importantly also in human right atrial preparations (Fig. 3A, B). Isoprenaline (10 nM) shortened the duration of contraction, and additionally applied OME prolonged duration of contraction (Fig. 3A). Moreover, in the presence of OME but not in its absence, Ca^2+^ increased the duration of single contractions (Fig. 3B).

### Effect of mavacamten-461 on time parameters of single contractions in electrically driven mouse left atrial preparations and electrically driven human right atrial preparations

**Fig. 4:**
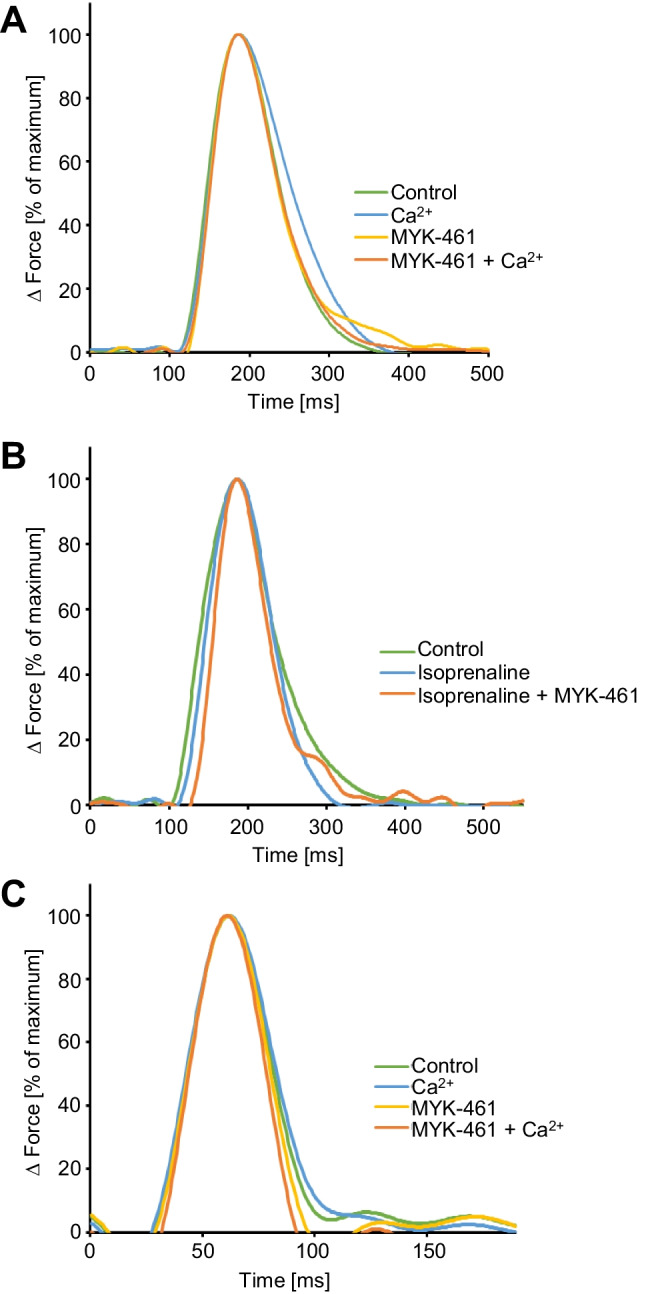
Effects of mavacamten-461 (MYK-461) on time parameters of contraction in human and mouse atrial preparations. MYK-461 shortens the duration of contraction. Ordinates: normalized force of contraction (% of maximum effect). Abscissae: time of contraction in milliseconds (ms). **A** After control contractions in human atrial preparations (Ctr), calcium ions (Ca^2+^) prolonged the duration of contraction, and additionally applied MYK-461 shortened duration of contraction. **B** In contrast, in human atrial preparations, isoprenaline shortened duration of contraction that was further shortened by MYK-461. **C** Similarly, in mouse left atrial preparations, Ca^2+^ prolonged duration of contraction alone, and additionally applied MYK-461 prolonged even more the duration of muscle contraction in the additional presence of Ca^2+^

Similar experiments such as in Fig. 3 were also performed for MYK-461 in order to facilitate a direct comparison of MYK-461 and OME. Interestingly, MYK-461 decreased time to peak tension and time of relaxation in mouse left atrial preparations (Fig. 4C). In contrast to OME, MYK-461 decreased time to peak tension and time of relaxation also in human atrial preparations (Fig. 4A, B). This shortening of muscle contraction started at 10 µM MYK-461, and additionally applied isoprenaline could not reduce time parameters further (Fig. 4B).

### Effect of omecamtiv mecarbil and mavacamten-461 on time parameters of contraction in electrically driven human right atrial preparations

**Fig. 5:**
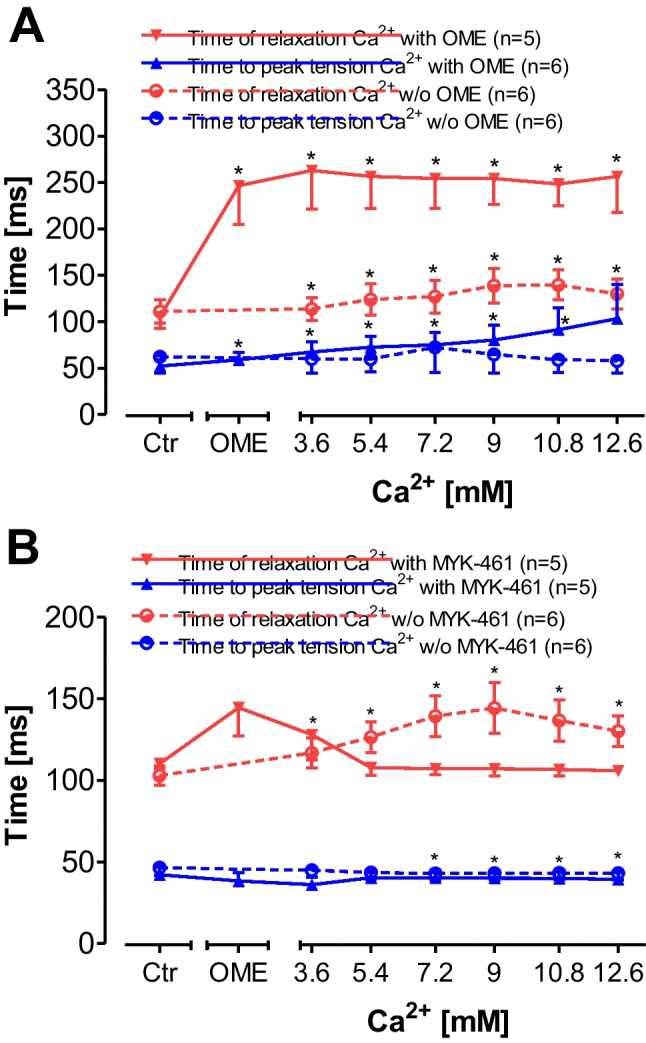
Concentration-dependent effects of calcium ions alone or in the presence of omecamtiv mecarbil (OME) or mavacamten-461 (MYK-461) on time to peak tension or time of relaxation in electrically stimulated human atrial preparations. Calcium ions (Ca^2+^) were cumulatively applied alone or in the presence of OME (**A**) or MYK-461 (**B**). Abscissae: concentration of calcium ions in millimolar concentrations (mM). Numbers in brackets indicate number of experiments. *p < 0.05 versus pre-drug value (Ctr) set as 100%

In the presence but not in the absence of OME, Ca^2+^ concentration- and time-dependently prolonged time to peak tension and relaxation time (Fig. 5A). Moreover, MYK-461 shortened Ca^2+^-induced prolongation of the duration of contraction (Fig. 5B).

### Effect of omecamtiv mecarbil and mavacamten-461 on velocity of contraction in electrically driven human right atrial preparations

**Fig. 6:**
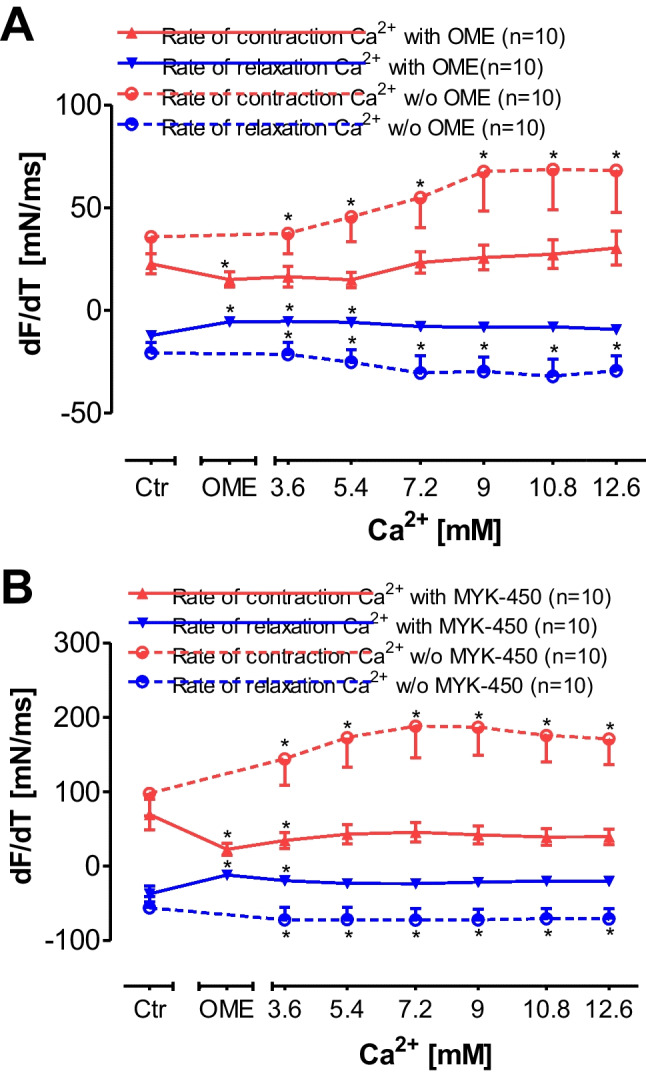
Effect of calcium ions (Ca^2+^) on the first derivate of force of contraction versus time in human right atrial preparations in the absence or presence of omecamtiv mecarbil (OME) or mavacamten-461 (MYK-461). OME (**A**) or MYK-461 (**B**) reduced Ca^2+^-induced increases in the rate of tension development (dF/dt: ordinates). Abscissae: concentration of calcium ions in millimolar concentrations (mM). Numbers in brackets indicate number of experiments. *p < 0.05 versus pre-drug value (Ctr) set as 100%

OME decreased dF/dt in relative values in a concentration- and time-dependent fashion (Fig. 6A), whereas these parameters were increased by a single concentration of 1 µM isoprenaline in the same preparations additionally applied (data not shown). Moreover, like OME, MYK-461 decreased dF/dt in absolute values in a concentration- and time-dependent fashion (Fig. 6B), whereas these parameters were increased by additionally applied isoprenaline in the same preparations (data not shown).

### Effect of omecamtiv mecarbil on force contraction in the absence and presence of calcium ions in the organ bath in electrically driven mouse left atrial preparations and electrically driven human right atrial preparations

**Fig. 7:**
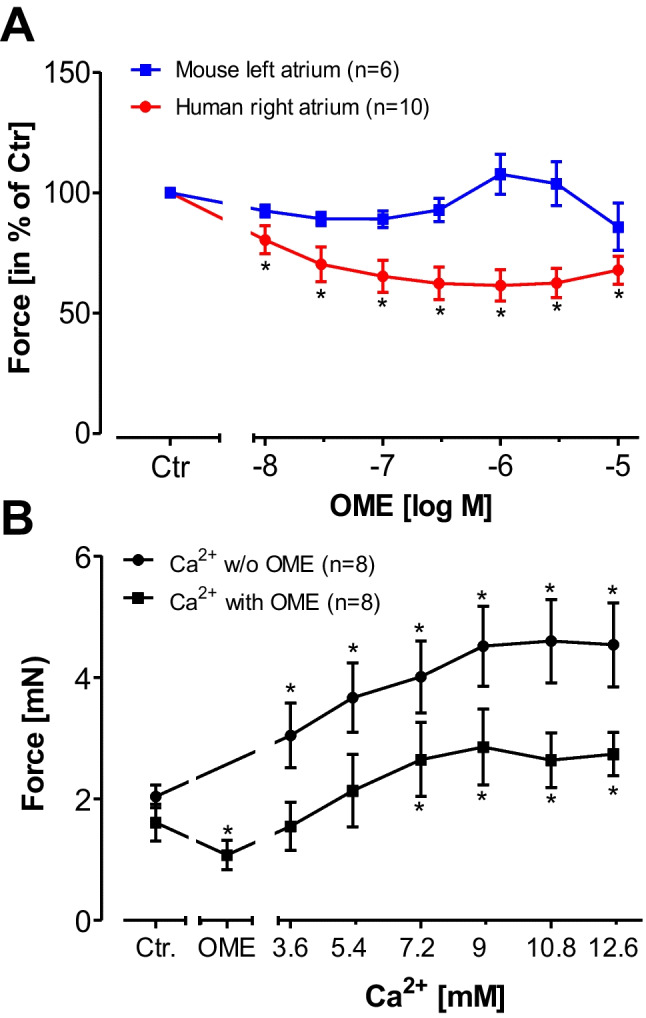
Omecamtiv mecarbil (OME) did not increase force of contraction in human atrium. **A** Effect of OME in isolated right atrial preparations from human hearts (red circles) or in isolated left atrial preparations from mice (blue squares). **B** Effect of calcium ions (Ca^2+^) alone (circles) or in the presence of OME (squares) on force of contraction in human right atrial preparations. Ordinates: force of contraction in % of pre-drug value (= control: Ctr) Abscissae: decadic logarithm of the concentrations of OME or millimolar (mM) concentrations of Ca^2+^. *p < 0.05 versus Ctr. Numbers in brackets indicate the number of experiments

OME did not increase force of contraction neither in human atrial preparations nor in mouse left atrial preparations (Fig. 7A). Subsequently applied isoprenaline (1 µM) increased force of contraction (data not shown). Ca^2+^ increased force of contraction in human atrial preparations, but these Ca^2+^ was neither more potent nor more effective in the presence of OME, arguing against a Ca^2+^-sensitizing effect of OME in the human heart (Fig. 7B).

### Effect of mavacamten-461 on force contraction in the absence and presence of calcium ions in the organ bath in electrically driven mouse left atrial preparations and electrically driven human right atrial preparations

**Fig. 8:**
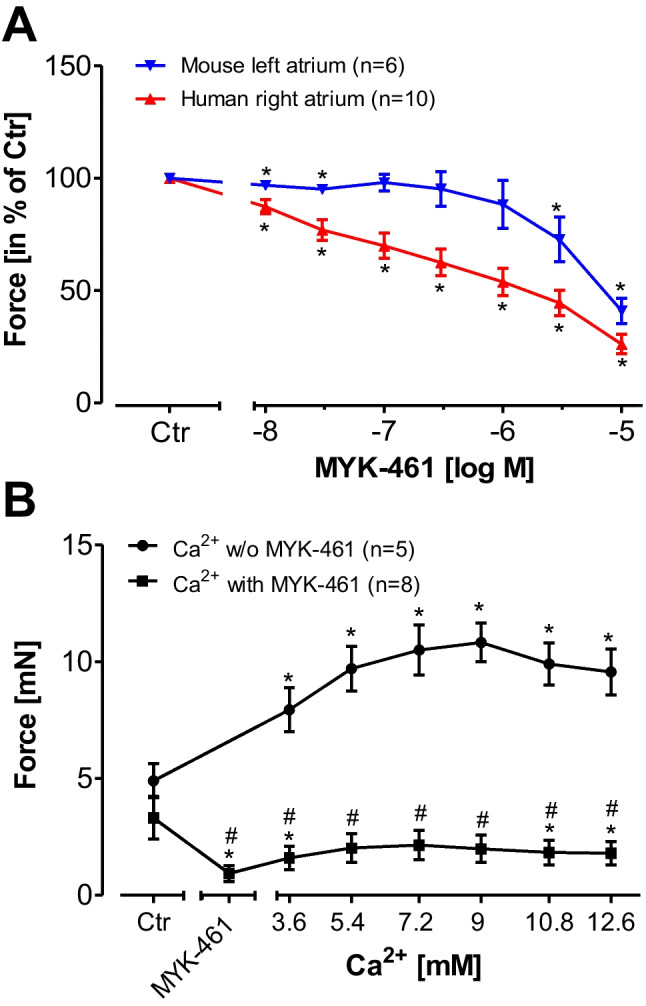
Mavacamten-461 (MYK-461) decreased force of contraction in human atrium. **A** Effects of MYK-461 in human right atrial preparations (red triangles) or mouse left atrial preparations (blue triangles). **B** Effects of calcium ions (Ca^2+^) alone (circles) or in the presence of 10 µM MYK-461 (squares) in isolated right atrial preparations from human hearts. Ordinates: force of contraction in % of pre-drug value (= control: Ctr.) Abscissae: decadic logarithm of the concentrations of MYK-461 or millimolar (mM) concentrations of Ca^2+^. *p < 0.05 versus Ctr. Numbers in brackets indicate the number of experiments

Quite different results were obtained with the MYK-461. MYK-461 concentration- and time-dependently reduced force of contraction in isolated electrically driven mouse atrial preparations (Fig. 8A). MYK-461 was even more potent to decrease force of contraction in human atrial preparations compared to mouse atrial preparations (Fig. 8A). Again, as a positive control, isoprenaline was given which increased force of contraction (data not shown). Ca^2+^ increased force of contraction in human atrial preparations, but these effects were nearly abolished in the presence of MYK-461 consistent with MYK-461 being a Ca^2+^-desensitizing agent in the human heart (Fig. 8B).

## Discussion

### Contractile data in mouse left atrium

OME and MYK-461 have been studied before in living mice and in skinned mouse muscle fibers. To the best of our knowledge, both compounds were never studied in spontaneously beating mouse right atrial preparations nor in isolated electrically driven (1 Hz) left atrial preparations under isometric conditions which is a novelty of this work.

The present data on OME in mouse left atrial preparations concur with previous data in rat papillary (Lookin et al. 2022). Lookin et al. (2022) also did not detect a positive inotropic effect of OME in rat papillary muscle, but OME prolonged the duration of the muscle contraction. Their data were in contrast to early data in rat cardiomyocytes. In the original description of OME (Malik et al. 2011), a reduction in contractility in isolated electrically stimulated rat ventricular cardiomyocytes was reported. One might be able to reconcile these opposing findings in the rat heart by noticing that auxotonic contractions in single cells were studied (Malik et al. 2011, Fülöp et al. 2021), whereas later isometric contractions in rat muscle strips were investigated (Lookin et al. 2022). Adding to the complexity of the literature are other apparent species differences: in canine ventricular cardiomyocytes under auxotonic conditions, an increase in cell shortening reminiscent of a positive inotropic effect was reported by several groups (Butler et al. 2015, Gao et al. 2020). However, a consistent observation irrespective of region, species, isolated cells, or muscle strips was that OME prolonged the duration of the muscle contraction. This prolonged duration of contraction can be explained by the observation that in skinned fibers from animal hearts (mouse: Malik et al. 2011, Mamidi et al. 2015, 2017, 2021), OME increased the potency of external Ca^2+^ to raise force of contraction. We have noted such prolongation of contraction duration with a pure Ca^2+^ sensitizer called CGP 48,506 in guinea pig, but this was accompanied by clear positive inotropic effect in human atrial preparations (Zimmermann et al. 1996).

A negative inotropic effect of MYK-461 in mouse and dog cardiac preparations has been reported before, and we thus confirm those observations but also extend them to isolated mouse left atrial and right atrial preparations. The negative inotropic effect can be explained by the observation that in skinned mouse ventricular fibers (atrium apparently was never studied), MYK-461 reduced the maximal response to Ca^2+^ as well as the affinity for Ca^2+^ as expected from a Ca^2+^ desensitizer (Awinda et al. 2021).

Some explanatory words on the interpretation of the data on force in right atrium seem to be in order. Physiologically the mouse atrium exhibits a negative staircase or “treppe” phenomenon: that is, in mouse atrial preparations, an increase in the beating rate leads to a negative inotropic effect, and a reduction of the beating rate leads to a positive inotropic effect. Thus, when reporting force of contraction in spontaneously beating right atrial preparations, one always has to keep in mind the accompanying changes in beating rate. However, this possible complication does not apply to the present data: we did not observe changes in beating rate due to OME or MYK-461. Hence, the negative inotropic effects of MYK-461 in right atrium and the contraction prolonging effects of OME in right atrium are very likely direct effects on the cardiomyocytes and not secondary effects due to changed beating rates. Thus, from these data, force of contraction responded in left and right atrium similarly to OME or MYK-461, at least in the mouse atrium.

Interestingly, Ca^2+^ concentration response curves in mouse left atria gave functional evidence for Ca^2+^-desensitizing effect of MYK-461 and support the data in skinned mouse cardiac fibers by other groups. However, we did not observe evidence for a sensitizing effect of OME to increasing concentrations of Ca^2+^ in contracting mouse left atrial preparations. This is in line with a lack of any positive inotropic effects of OME alone in mouse atrium. One might argue that this might be due to different isoforms of myosin in rat and mouse heart. This interpretation is hardly convincing: in rat and mouse, the alpha myosin isoform of myosin is predominant. Moreover, in rat heart itself based on experimental conditions, a positive inotropic (Malik et al. 2011) or a negative inotropic effect of OME (Lookin et al. 2022) has been reported. Thus, experimental conditions seem to be important. It is noteworthy that positive inotropic effects to OME have hitherto only been noted under auxotonic conditions in single cardiomyocytes for instance in canine myocytes (Gao et al. 2020) or rat myocytes (Malik et al. 2011).

In isolated heart preparations, effects of OME on beating rate have apparently not yet been reported. This piece of information is helpful, because data on the effect of OME on beating rate in living animals has been reported. We reported for a pure Ca^2+^-sensitizer CGS that it did not alter beating rate in guinea pig right atrial preparations (Zimmermann et al. 1996) supporting our present data. The fact that MYK-461 did not alter beating rate in right atrial preparations might be regarded as an extension of this observation and might be interpreted to indicate that Ca^2+^ binding to the myofilaments does not impact the cardiac “clock” in the sinus node.

### Effects of omecamtiv mecarbil and mavacamten-461 in human atrium

In agreement with one previous publication in human atrial and ventricular preparations, OME failed to increase force of contraction in human atrium (Dashwood et al. 2021). Like those authors, we noted prolonged duration of muscle contraction in human right atrial preparations by added OME. To the best of our knowledge, this is the first report on the reduction of the rate of force development and the rate of relaxation (dF/dt) in human atrial preparations. It is plausible that the prolongation of muscle contraction is due to Ca^2+^ sensitization: in skinned fibers of muscle from failing and non-failing human heart, less Ca^2+^ was required in the extracellular space to increase force half maximally in the presence than in the absence of Ca^2+^. Somewhat astonishingly, this sensitization did not lead to an increase in force of contraction. In contrast, we had reported for a pure Ca^2+^ sensitizer (that did not inhibit phosphodiesterases) that sensitized skinned human cardiac fibers (CGP 48,506) that this compound increased force of contraction in human ventricular muscle strips under conditions that were identical to that of this study (Zimmermann et al. 1998). Thus, our methods are sensitive enough to detect a positive inotropic effect of a Ca^2+^ sensitizer if such an effect exists. Thus, we must conclude that the biochemical mode of Ca^2+^ sensitization of OME is different from that of CGP 48,506, which is a plausible conclusion. On the other hand, like CGP 48,506, also OME prolonged the duration of muscle contraction, which concurs with a Ca^2+^-sensitizing effect of OME. Because OME does not increase force of contraction but prolongs muscle contraction time, it is expected that the rate of force generation and rate of relaxation are decreased, because not more time is available for the same amplitude of muscle contraction to be achieved. Often the rate of tension development is regarded as the gold standard for positive inotropy (this definition and other definitions of inotropy are discussed in Ahmad et al. 2019). Hence, a reasonable interpretation from our data is that OME is a negative inotropic compound and not a positive inotropic compound, in the mouse and human atrium. Hence, we suggest to reclassify OME. It is therefore unlikely that the beneficial effects of OME in clinical trials (Teerlink et al. 2016, 2020) are due to its inotropic support of the failing heart. We argue here that the mechanism(s) for the beneficial clinical effect of OME still need(s) to be elucidated. In a clinical trial with OME, beating rate slightly declined, systolic ejection time increased, and stroke volume went up (Teerlink et al. 2016). In patients with heart failure and a reduced ejection, OME had a lower composite end point of heart failure or death from cardiovascular (Teerlink et al. 2020). However, all-cause mortality or cardiovascular mortality was not reduced by OME in the trial (Teerlink et al. 2020). This unfavorable outcome has been hypothetically explained by a reduced diastolic filling time due to OME in the left ventricle and increased oxygen demand of myosin in the ventricle in the presence of OME (Komamura 2021). Others criticized that the study (Teerlink et al. 2020) was performed with only 2.5% of the patients taking SGLT2 inhibitors which are now strongly recommended in systolic heart failure guidelines because they reduce mortality, and therefore the study findings remained inconclusive because in the presence of SGLT2 inhibitors, no additional effect of OME might be noticeable, and at least the study design was in this regard outdated and would not help in directing nowadays the optimal drug treatment (Bellumkonda 2021). Others raised concern that OME in the trial (Teerlink et al. 2020) raised plasma troponin I levels, a marker of myocardial damage and a predictor of poor cardiac prognosis in heart failure patients (Bonapace and Molon 2021).

Interestingly, Ca^2+^ concentration response curves in human atrial preparations gave functional evidence for Ca^2+^-desensitizing effect of MYK-461 and support the data in skinned human cardiac fibers (non-failing human heart fibers: Awinda et al. 2020). The affinity of myofilaments to Ca^2+^ and the maximum force generation in skinned human ventricular fibers were reduced by MYK-461 (Awinda et al. 2020). However, we did not observe any evidence for a sensitizing effect of OME to increasing concentrations of Ca^2+^ in contracting mouse or human atria. This is in line with a lack of positive inotropic effects of OME alone in human atrium in sharp contrast with Ca^2+^-sensitizing effects of OME in skinned human fibers. We would argue here that it is an important finding of the present work that one cannot directly extrapolate from findings with drugs in skinned fibers to the situation in contracting tissue.

We hypothesize that this discrepancy might result from permeability problems: it is conceivable that OME acts indeed as a Ca^2+^ sensitizer if the sarcolemma is broken but cannot pass the intact sarcolemma. This hypothesis could be tested, for instance, with radioactive OME. However, we would argue that the fact that we detected prolonged contraction duration in our human samples is strong evidence that OME could get access to the myofilaments in our procedures. Another explanation which we currently favor is that OME has effects on other proteins in the sarcolemma (or beyond) that cancel the positive inotropic effect of OME on the myofilaments. Using radioactive OME, one could try to identify OME binding proteins in human hearts as a first experimental step in this direction. From a practical clinical perspective, our data and those of others (Dashwood et al. 2021) convincingly show that OME is not useful in a setting where mainly inotropic support is needed in a patient. Hence, the clinical usefulness of OME needs to be studied in more detail, and it remains to be seen whether OME will gain widespread use in hospitals.

In contrast, our contractile data make it easy to understand clinical benefits of MYK-461. It is well established that in HOCOM, the symptoms of the patient are improved if negative inotropic compounds are given. Such compounds included in the past verapamil, a drug that can block cardiac L-type Ca^2+^ channels and thus reduce the availability of Ca^2+^ near the filament and thus reducing force of contraction. In this pattern, one can understand the role of MYK-461 which requires more Ca^2+^ in skinned human cardiac fibers for half maximal contraction. To the best of our knowledge, this is the first report where a negative inotropic effect of MYK-461 in any human cardiac preparation is reported. In line with the reduced force of contraction by MYK-461 in atrial preparations, it is plausible that Ca^2+^ desensitization of muscle fibers underlies the reduced contraction times we observed. Indeed, our data on Ca^2+^ force relationships in the presence of MYK-461 point into that direction: the combination of reduced contraction times and the reduced force generation by MYK-461 easily explains why mavacamten reduced rate of contraction and relaxation in our study in human (and mouse) preparations. Hence, our data support a clinical utility of MYK-461 in the rare cases where heart failure results from a hyperdynamic contraction of the heart (in animals like some strains of cats and humans with genetic mutations of myosin). Thus, MYK-461 is a small step forward towards a personalized drug therapy of heart failure. It still remains to be shown that mavacamten reduces mortality in any group of patients before it can be widely prescribed.

An apparent limitation of our work lies in the fact that we investigated drugs intended for heart failure therapy in human atrial preparations and not in ventricular preparations. Unfortunately, in our institution, we do not have access to human ventricular tissue. Likewise, we do rarely get atrial tissue from failing hearts. Usually, we only receive atrial tissue from bypass surgery of patients who are not (yet) in heart failure. Therefore, we have restricted our efforts to that tissue that was available to us.

In summary, the present work confirms that OME does not increase force of contraction but prolongs duration of single contractions in isolated human atrial preparations. We extend these results for OME to left atrial preparations from wild-type mice. We noticed a negative inotropic effect of MYK-461 in isolated left atrial preparations from wild-type mice and in isolated human atrial preparations.

## Supplementary Information

Below is the link to the electronic supplementary material.

Supplementary file1 (PZF 201 kb)

Supplementary file2 (PZF 202 kb)

Supplementary file3 (PZF 191 kb)

Supplementary file4 (PZF 221 kb)

Supplementary file5 (PZF 174 kb)

Supplementary file6 (PZF 182 kb)

Supplementary file7 (PZF 176 kb)

Supplementary file8 (PZF 184 kb)

## Data Availability

The data of this study are available from the corresponding author upon reasonable request.
